# Non-vernalization requirement in Chinese kale caused by loss of *BoFLC* and low expressions of its paralogs

**DOI:** 10.1007/s00122-021-03977-x

**Published:** 2021-10-29

**Authors:** Qiwei Tang, Hanhui Kuang, Changchun Yu, Guanghui An, Rong Tao, Weiyi Zhang, Yue Jia

**Affiliations:** Key Laboratory of Horticultural Plant Biology, Ministry of Education, Key Laboratory of Horticultural Crop Biology, College of Horticulture and Forestry Sciences, Huazhong Agricultural University, Wuhan, 430070 China

## Abstract

**Key message:**

**We identified the loss of** ***BoFLC*** **gene as the cause of non-vernalization requirement in** ***B. oleracea***. **Our developed codominant marker of** ***BoFLC*** **gene can be used for breeding program of** ***B. oleracea*** **crops**.

**Abstract:**

Many species of the Brassicaceae family, including some Brassica crops, require vernalization to avoid pre-winter flowering. Vernalization is an unfavorable trait for Chinese kale (*Brassica oleracea* var. *chinensis Lei*), a stem vegetable, and therefore it has been lost during its domestication/breeding process. To reveal the genetics of vernalization variation, we constructed an F_2_ population through crossing a Chinese kale (a non-vernalization crop) with a kale (a vernalization crop). Using bulked segregant analysis (BSA) and RNA-seq, we identified one major quantitative trait locus (QTL) controlling vernalization and fine-mapped it to a region spanning 80 kb. Synteny analysis and PCR-based sequencing results revealed that compared to that of the kale parent, the candidate region of the Chinese kale parent lost a 9,325-bp fragment containing *FLC* homolog (*BoFLC*). In addition to the *BoFLC* gene, there are four other *FLC* homologs in the genome of *B. oleracea*, including *Bo3g005470*, *Bo3g024250*, *Bo9g173370,* and *Bo9g173400*. The qPCR analysis showed that the *BoFLC* had the highest expression among the five members of the *FLC* family. Considering the low expression levels of the four paralogs of *BoFLC*, we speculate that its paralogs cannot compensate the function of the lost *BoFLC,* therefore the presence/absence (PA) polymorphism of *BoFLC* determines the vernalization variation. Based on the PA polymorphism of *BoFLC*, we designed a codominant marker for the vernalization trait, which can be used for breeding programs of *B. oleracea* crops.

**Supplementary Information:**

The online version contains supplementary material available at 10.1007/s00122-021-03977-x.

## Introduction

Chinese kale (*Brassica oleracea var. chinensis Lei*) is a Brassica vegetable widely planted in Southern China and Southeast Asia (Lei et al. 2017; Qian et al. 2016). The stem and leaves of Chinese kale are popularly consumed owing to its flavors, vivid colors, rich anticarcinogenic and antioxidative components such as vitamin C, total phenolics, carotenoids, and glucosinolates (Sun et al. 2011, 2012; Wei et al. 2011; Wu et al. 2017). The bolting stems of Chinese kale are generally harvested and consumed, and consequently, Chinese kale has been bred for early bolting without vernalization.

For plants, the timing of reproduction is mainly governed by flowering time regulation. In temperate climates, two most important cues to induce flowering are a period of extended cold (also known as the vernalization pathway) and day length (the photoperiod pathway) (Blumel et al. 2015). The vernalization pathway is a complex regulatory system associated with transcriptional and epigenetic regulations (Song et al. 2013). In Arabidopsis, the vernalization pathway promotes flowering in response to extended exposure to low temperatures (Searle et al. 2006). The natural variation of flowering time in Arabidopsis can be explained by two loci, *FRIGIDA* (*FRI*) and *FLOWERING LOCUS C* (*FLC*) (Burn et al. 1993; Clarke and Dean 1994; Johanson et al. 2000; Koornneer et al. 1994; Michaels and Amasino 1999; Sheldon et al. 1999). *FLC* encoding a MADS-box transcription factor inhibits expression of the central flowering regulator *FLOWERING LOCUS T* (*FT*) (Sheldon et al. 2000). FLC also binds to the promoters and represses the expressions of several other important flowering genes, such as *FLOWERING LOCUS D* (*FD*), *SUPPRESSOR OF OVEREXPRESSION OF CONSTANS 1* (*SOC1*), and *TEMPRANILLO 1* (*TEM1*) (Deng et al. 2011; Searle et al. 2006). *FRI* and *VERNALIZATION INSENSITIVE 3* (*VIN3*) are two critical genes regulating the expression of *FLC* (Johanson et al. 2000; Michaels and Amasino 1999; Sheldon et al. 1999). *FRI* is responsible for a high production of *FLC* and *VIN3* genes at both protein and transcriptional levels during vernalization. The vernalization process overrides the FRI-mediated control of *FLC*, resulting in the repression of transcriptional activity of *FRI* and the promotion of flowering initiation (Mempel et al. 2004; Sheldon et al. 2000).

The main vernalization components are conserved in Brassicaceae species, and their paralogs also have similar functions in regulating flowering time. Variation in paralogs of *FLC* has been reported to be related to flowering time in *B. rapa*, *B. napus*, and *B. oleracea* (Hou et al. 2012; Irwin et al. 2016; Okazaki et al. 2007; Osborn et al. 1997; Pires et al. 2004; Razi et al. 2008; Ridge et al. 2015; Schiessl et al. 2017; Tadege et al. 2001; Wu et al. 2012; Yuan et al. 2009; Zhao et al. 2010). There are four homologs of *FLC* in *B. rapa*, including *BrFLC1*, *BrFLC2*, *BrFLC3*, *BrFLC5*. A naturally occurring InDel variation in *BrFLC2* and a splicing site mutation in *BrFLC1* have also been found to contribute to flowering time variation in *Brassica rapa* (Wu et al. 2012; Yuan et al. 2009). A study based on genetic mapping and QTL analysis has demonstrated that *BrFLC1*, *BrFLC2,* and *BrFLC5* play important roles in vernalization, suggesting that these *FLCs* control flowering response in a dosage-dependent manner (Schranz et al. 2002b). Rapeseed (*Brassica napus*) with a highly duplicated genome carries nine copies of *Bna.FLC,* and insertion of a Tourist-like MITE into the upstream region of the *BnFLC.A10* gene affects vernalization requirement (Hou et al. 2012). Four *FLC* homologs (*BoFLC1*, *BoFLC3*, *BoFLC4*, *BoFLC5*) have been identified from *B. oleracea* (Lin et al. 2005; Schranz et al. 2002b). *BoFLC.C2* co-located with a flowering time QTL has been reported to be responsible for the late flowering trait in broccoli (*Brassica oleracea var. italica*) (Okazaki et al. 2007; Zhao et al. 2010).

Non-vernalization is an important agronomic trait for Chinese Kale, a vegetable with bolting stem harvested and consumed. In this study, we explored the genetic mechanism underlying vernalization variation in Chinese Kale and kale based on BSA + RNA-seq. The candidate gene was identified by synteny analysis of the candidate region and further verified through the comprehensive analysis of its paralogs. This study provides insight into the genetic and molecular mechanisms of vernalization in *B. oleracea*. The identified genetic variations can be used in marker-assisted genetic breeding of *B. oleracea* crops.

## Materials and methods

### Materials used in this study

The *B. oleracea* var. *Alboglabra* and *B. oleracea* var. *acephala* were used as parents to generate segregating population in this work. Cultivars, parental lines, F_1_ hybrid, F_2_, and advanced generations were grown in the field on the campus of Huazhong Agriculture University, Wuhan, China. The seeds of *B. oleracea* cultivars were acquired from National Center for Vegetable Improvement (Central China) or bought from commercial websites of JingDong (https://www.jd.com/).

### Identification of vernalization variation and data analysis

F_1_ plants were self-pollinated to produce F_2_ population. A total of 107 individuals in F_2_ population were planted in September, 2016 in Wuhan, China. Flowering status was investigated on December 27, 2016. The plants flowering before December 27, 2016 were defined as non-vernalization plants, and the plants flowering after December 27, 2016 were designated as vernalization plants. Three F_3_ families were planted in September, 2017 in Wuhan, China. Flowering status was investigated in January 2018. One F_4_ family (generated from one of the three F_3_ families) with 268 individuals was planted in March, 2018. Flowering status was investigated in May, 2018.

ANOVA and phenotypic variation explained (PVE) analysis were conducted using R package 'car' (version 3.0–11) (https://CRAN.R-project.org/package=car).

### Bulked segregant analysis (BSA) and RNA-seq

BSA in combination with RNA-seq (BSR) was used to identify QTL controlling flowering time following the procedures reported previously (Yu et al. 2020). In this study, the leaves from 20 four-month-old early flowering plants in an F_2_ segregating population were mixed as an early flowering pool, and the leaves from 20 late flowering plants were mixed as late flowering pool. Total RNA was extracted from the two pools using TransZol reagent (TRANSGEN, Beijing, China). RNA-seq was performed on Illumina Hiseq2500 platform, and approximately 6 Gb clean data were obtained from each pool. Clean data were aligned to the *B. oleracea* genome TO1000 V2.1 (Liu et al. 2014) using Bowtie software (version 1.3.0) (Langmead et al. 2009). SNP calling was performed using SAMtools (version 1.7) (Li et al. 2009). Low-quality SNPs with mapping quality value < 30, read depth < 10, or base quality value < 20 were excluded. SNP-index was used to identify the target region for early/late flowering trait. SNP-index was calculated by subtracting the allelic SNP frequency of the early flowering pool from that of the late flowering pool. Average SNP-index was calculated using a 900 kb sliding window with a step size of 400 kb, and was plotted along the nine chromosomes of *B. oleracea*. Finally, under the null hypothesis of no QTL, the 95% statistical confidence intervals of SNP-index were calculated as described previously (Takagi et al. 2013). In the plot, a peak may represent a region harboring a gene related to flowering time. The cleaved amplified polymorphic sequence (CAPS) markers were designed in the candidate region, and were used to screen the population and obtain causal genes by fine mapping.

### PCR amplification of a large fragment

The genomic DNA of the parent plants was extracted from leaves using cetyltrimethylammonium bromide (CTAB) method (Porebski et al. 1997). Each 50 μL PCR mixture contained 1 μL of DNA template (100 ng/μL), 1 μL of each primer (10 μM), 22 μL of ddH_2_O, and 25 μL PrimeSTAR GXL Premix (Takara Bio, Japan). The PCR program included 32 cycles of 98 °C for 10 s, 57 °C for 15 s, and 68 °C for 10 min. The PCR products were analyzed using 1% agarose gel electrophoresis. Purified PCR fragments were sequenced by commercial company Tsingke Biotechnology (Wuhan, China) through primer-walking method.

### Sequence analysis

We analyzed the syntenic regions of the genome in *B. oleracea* species (the ‘TO1000’ reference genome, version 2.1) (Liu et al. 2014) and other species in Brassicaceae, including *B. rapa* (the ‘Chiifu-401-42’ reference genome, version 1.5) (Wang et al. 2011), *B. napus* (the Darmor-bzh reference genome, version 4.1) (Chalhoub et al. 2014), and Arabidopsis (version TAIR11) (Garcia-Hernandez et al. 2002) using BLAST (version 2.11.0) (Buchfink et al. 2015) with parameter “blastp -e 10–10”. Primers used in this study were designed using PRIMER3 (version v.0.4.0) (Untergasser et al. 2012) (supplementary dataset 3). GENEIOUS (version 7.0.9; Biomatters Ltd, Auckland, New Zealand) was used to analyze sequence divergence among *FLC* homologs in *B. oleracea*.

### Construction of phylogenetic tree

We obtained *FLC* gene identities (IDs) from previous studies of Brassicaceae (Calderwood et al. 2021; Lin et al. 2005; Schranz et al. 2002a). The amino acid sequences of the *FLC* homologs in different species were retrieved from EnsemblPlants database (http://plants.ensembl.org/index.html) using *FLC* gene IDs. The protein sequences were aligned using Clustal X (version 2.1) (Larkin et al. 2007). A maximum-likelihood phylogenetic tree was constructed using MEGA 7.0 (Kumar et al. 2016). Bootstrapping was performed with 1000 replications.

### Gene expression analysis

Total RNA was extracted from leaves using TransZol reagent (TRANSGEN, China, Beijing) following the manufacturer’s instructions. Total RNA was treated with DNase I (Thermo, Waltham, USA) to remove the contaminated genomic DNA. The cDNA was synthesized using TransScript cDNA Synthesis SuperMix (TRANSGEN, Beijing, China). All reactions were performed using ChamQ SYBR qPCR Master Mix (Vazyme biotech, Nanjing, China) with a reaction system containing 10.0 μL of 2 × ChamQ SYBR qPCR Master Mix, 0.4 μL of primers, 1.0 μL of cDNA, and 8.2 μL of ddH_2_O. Quantitative real-time PCR (qRT-PCR) was conducted on QuantStudioTM 6 Real-Time PCR System (Thermo). The reactions were performed with three biological replicates and three technical replicates. The transcript levels of genes in different individuals were analyzed using the 2^−ΔΔCT^ method and normalized with *actin* as an internal control. (Livak and Schmittgen 2001). The primers used for qRT-PCR assays were listed in the supplemental dataset 3.

## Results

### Genetic analysis of flowering time using BSA in combination with RNA-seq (BSR)

**Fig. 1:**
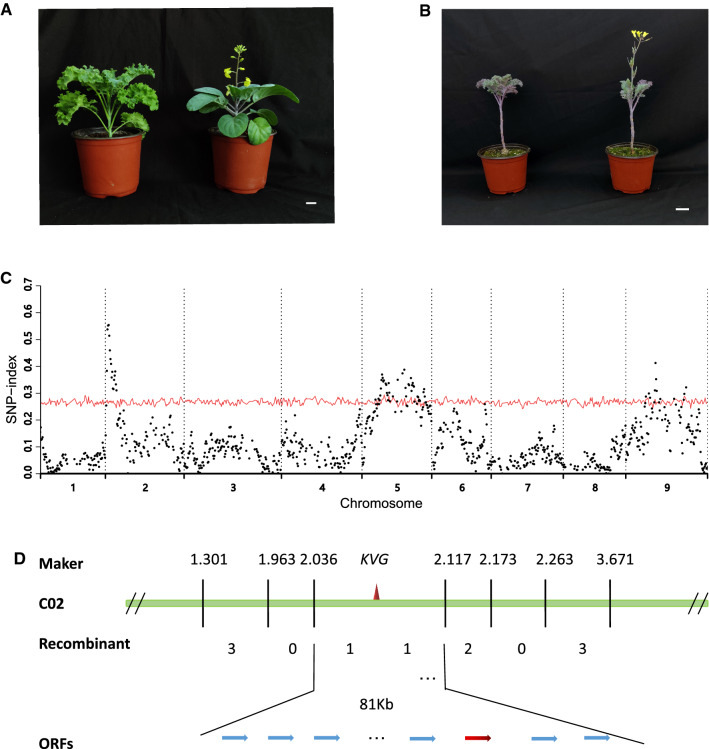
Genetic mapping of genes affecting flowering time. **A** Parents used for constructing the F_2_ population. Kale, left panel; Chinese kale, right panel. Bar = 2 cm. **B** Flowering individual and non-flowering individual in an F_4_ family. Bar = 2 cm. **C** Plot of SNP-index along 9 chromosomes of cabbage. The *x*-axis represents the nine chromosomes and the y-axis represents the average SNP-index in each sliding window. The red curves represent statistical confidence intervals under the null hypothesis of no QTL at *p* < 0.05. **D** Fine mapping of *KVG1*. The numbers below the horizontal line and between two markers refer to the number of recombinants from 587 individuals

As a vegetable with bolting stem harvested and consumed, Chinese kale has been bred for early bolting. In contrast, kale, either as a vegetable or ornamental crop, requires vernalization. To genetically dissect flowering time variations between the two closely related crops, a Chinese kale cultivar was crossed with a kale cultivar (Fig. 1A), and the F_1_ hybrids were selfed to generate an F_2_ segregating population. BSR method was used to identify QTL controlling flowering time, and several peaks were observed in the plot. Each peak was considered to represent a QTL controlling flowering variation in F_2_ population (Fig. 1C).

We named the QTL on chromosome 2, 5, and 9 as *Kale Vernalization Gene 1–3* (*KVG1-3*), respectively. ANOVA indicated no interactions among the three loci (*p* = 0.47, 0.86, 0.88), and *KVG1, KVG2* and *KVG3* explained 18.44%, 6.30%, and 2.08% of vernalization phenotypic variation, respectively. We concluded that in the F_2_ segregating population, the vernalization was a quantitative trait controlled by at least three loci, and that *KVG1* was a major QTL for vernalization (Fig. 1C).

### Construction of single-gene segregating population for vernalization trait

To clone the major QTL *KVG1* located on chromosome 2, we selected several F_2_ individuals which are heterozygous at *KVG1* and homozygous at other QTL. These individuals were selfed to generate F_3_ families in which only *KVG1* controls flowering time variation. The phenotype analysis of three F_3_ families showed that flowering time was a qualitative trait in F_3_ families, either flowering or non-flowering before the winter, In January 2018, 78 individuals flowered and 231 individuals did not flower with a ratio of 1:3 (*χ*^2^ = 0, *p* = 1). The late flowering plants did not blossom until March 2018, showing typical vernalization characteristic. To further test whether the flowering trait was consistent with vernalization, we planted one F_4_ family (generated from one of the three F_3_ families) with 268 individuals in March, 2018. Of them, 73 individuals flowered in May, 2018, whereas 195 individuals did not flower until March 2019 with a ratio of 1:3 (*χ*^2^ = 0.01, *p* = 0.92), suggesting that the requirement for vernalization was determined by a single gene (*KVG1*) with complete dominance.

### Fine mapping of *KVG1*

To fine map the *KVG1* gene, we designed several genetic markers in the target region on chromosome 2 based on the RNA-seq data (supplemental dataset 3). The 587 individuals from the segregating populations were genotyped, respectively using the far-end marker C02-1.301 and C02-3.671 to identify recombinants (supplemental dataset 3) (Fig. 1D). The recombinants were further genotyped using the other five markers in the target region (Fig. 1D). Consequently, *KVG1* was mapped between markers C02-2.036 and C02-2.117, spanning a region of approximately 80 kb (Fig. 1D).

### *FLC* gene as the candidate gene of *KVG1*

**Fig. 2:**
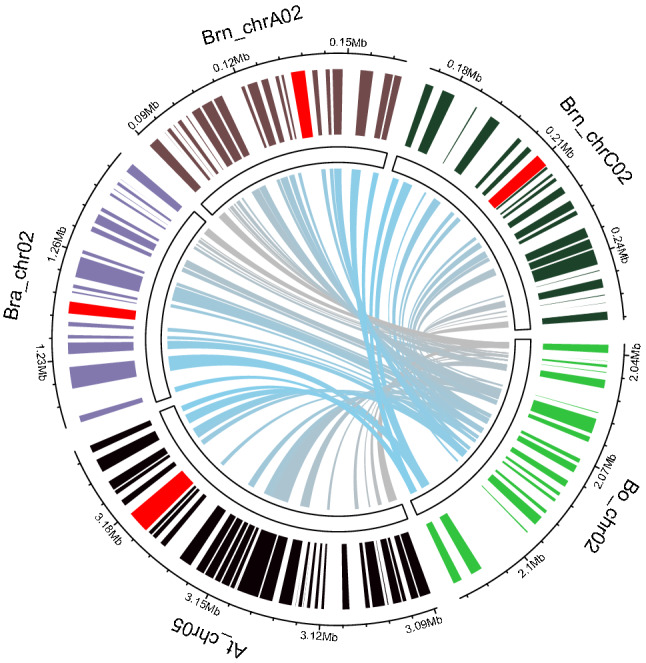
Synteny analysis of the *KVG1* candidate region in different Brassica species and Arabidopsis. Each block on the outside layer presents the positions of genes in the synteny region, and the red blocks indicate *FLC* genes in the corresponding genomes. Bo_chr02, Bra_chr02, Brn_chrA02, Brn_chrC02, and At_chr05 represent the chromosomes of *B. oleracea*, *B. rapa*, *B. napus*, *B. napus*, and Arabidopsis, respectively. Linking lines inside the circle represent synteny pairs between two species

The 80 kb candidate region contained 17 genes in cabbage reference genome (TO1000 V2.1) (Liu et al. 2014), but none of these genes are associated with flowering time according to their annotations (supplemental dataset 1) (Liu et al. 2014). We used these 17 genes to search for syntenic regions of other genomes in the species of Brassicaceae, including *B. rapa*, *B. napus,* and Arabidopsis. Interestingly, one *FLC* homolog was localized in the syntenic region of the genomes in *B. rapa*, *B. napus,* and Arabidopsis (Fig. 2).

**Fig. 3:**
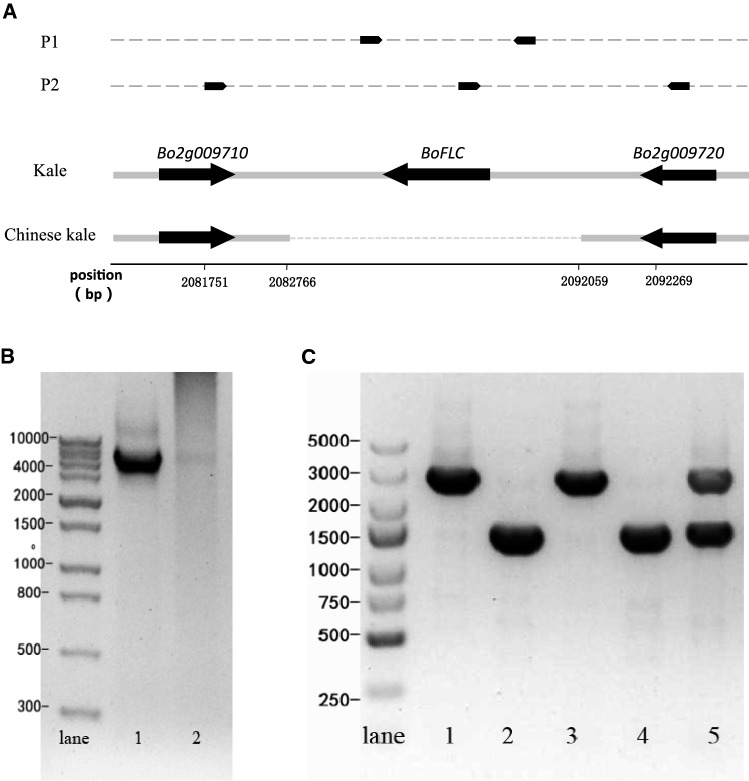
Deletion at the *KVG1* locus. **A** Positions of genes and primers in kale and Chinese kale. **B** Long PCR products amplified with P1 primers. lane 1, kale parent; lane 2, Chinese kale parent. **C** Codominant PCR products amplified with three primers act as a marker to detect the deletion of *BoFLC*. lane 1, the kale parent; lane 2, the Chinese kale parent; lane 3, Early flowering plant with homozygous genotype at the *KVG1* locus from the segregating population; lane 4, Late flowering plant with homozygous genotype at the *KVG1* locus from the segregating population; lane 5, Late flowering plant with heterozygous genotype in the segregating population

Although no *FLC* homolog was detected in the reference genome of *B. oleracea*, we speculated that there might be *FLC* homolog in the candidate region of kale but not in that of Chinese kale. To test this speculation, a pair of primers located in the two flanking genes *Bo2g009720* and *Bo2g009710* were designed and were used to amplify PCR products from kale and Chinese kale (Fig. 3A). The results showed that the fragments of approximately 1 kb and 10 kb were amplified from Chinese kale and kale, respectively. The two amplified PCR products were sequenced, and 9,325 bp was found to be deleted from Chinese kale. In the deleted region, one 3,284-bp sequence encodes an FLC homolog. We referred to the *FLC* homolog as *BoFLC* hereafter*.* A pair of primers flanking the *BoFLC* (P1) were designed (Fig. 3A), and using this pair of primers, intact *BoFLC* was amplified from the kale parent (Fig. 3B).

Subsequently, we designed three primers located in *Bo2g00917*, *BoFLC* and *Bo2g009720*, respectively (Fig. 3A). These three primers were simultaneously used for PCR reaction to generate a codominant marker (Fig. 3C). This resultant codominant marker co-segregated with the vernalization trait in the 587 individuals. Therefore, we showed genetically that vernalization was not required in Chinese kale due to the deletion of a 9,325-bp fragment containing the *BoFLC* gene.

### Sequence divergence among *FLC* homologs in *B. oleracea*

**Fig. 4:**
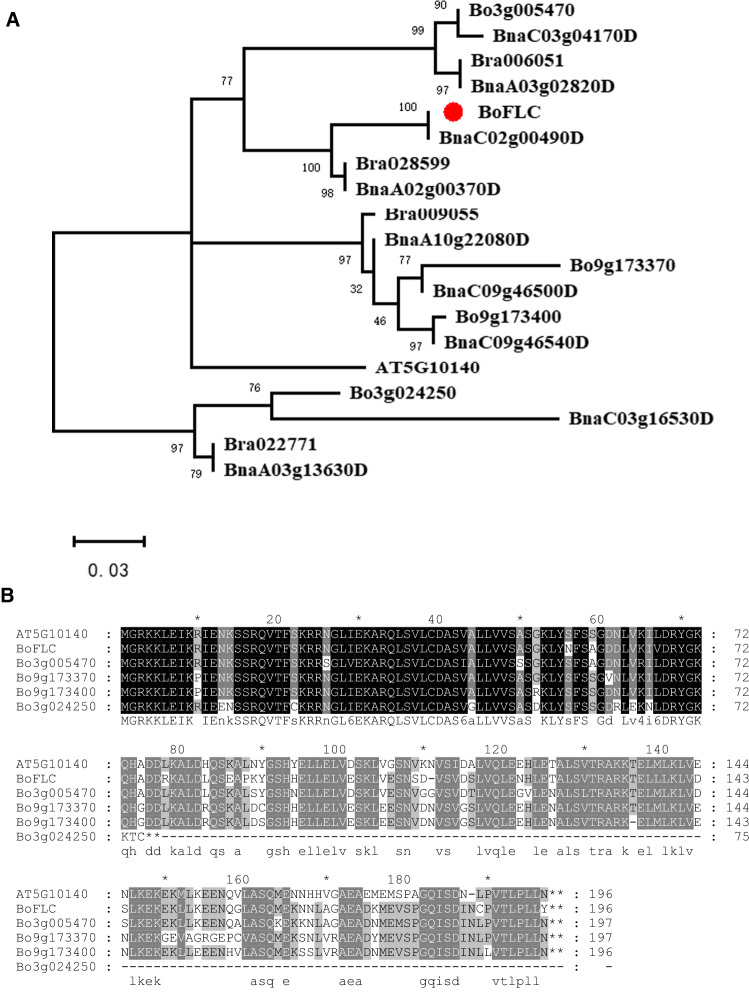
Sequence analysis of *FLC* homologs in Brassicaceae. **A** Phylogenetic tree of *FLC* homologs. Prefixes Bo, Br, Bna, and AT represent *FLC* homologs from *B. oleracea*, *B. rapa*, *B. napus,* and Arabidopsis, respectively. **B** Alignment of protein sequences of *FLC* homologs in *B. oleracea* and Arabidopsis. Shade indicates perfectly (black), highly (≥ 80%, dark grey), moderately (≥ 60%, grey) conserved residues

In addition to *BoFLC*, there existed four other *FLC* homologs in the genome of *B. oleracea*, including *Bo3g005470*, *Bo3g024250*, *Bo9g173370* and *Bo9g173400*. Phylogenetic analysis was carried out to explore the relationship between *FLC* homologs in the Brassicaceae species, including *B. oleracea*, *B. rapa*, *B. napus*, and Arabidopsis*.* The results revealed that the protein sequences of the orthologs/homeologs were more closely related to each other than those of the paralogs within a genome (Fig. 4A), suggesting that the *FLC* homologs were duplicated before the speciation of Brassica species, which was consistent with the previous study (Schranz et al. 2002b).

Our manual annotation of the *FLC* homologs in the genome of *B. oleracea* revealed that a 1-bp insertion in the second exon of *Bo3g024250* led to frame shift and early termination. Since this 1-bp insertion also occurred in both the kale and Chinese kale parents, this gene was inferred to have no effect on flowering time variation between the two parents. Therefore, this gene was excluded from the subsequent investigation. The coding sequences of the other four *FLC* genes (excluding *Bo3g024250*) exhibited 76.0–90.9% nucleotide identity and 84.2–97.8% amino acid identity. Protein sequences of these FLC homologs were highly conserved (Fig. 4B). In contrast to those of their highly conserved coding regions, the sequences of promoter regions of these four *FLC* homologs exhibited low nucleotide identity (less than 48.7%).

In order to investigate the genetic variations of the other three *FLC* genes, we designed PCR primers specific to each *FLC* homolog to amplify their sequences from the kale and Chinese kale parents (supplemental dataset 3). The *Bo9g173370* gene had identical coding sequences between the two parents, but this gene exhibited one 10-bp InDel in intron 4, and one 21-bp InDel, and four SNPs in the promoter region. The *Bo9g173400* gene was identical between the two parents. The *Bo3g005470* gene displayed one SNP in the coding sequence and six indels and multiple SNPs in introns. In summary, the presence/absence (P/A) of the *BoFLC* gene on chromosome 2 is the main difference in the *FLC* family between the kale and Chinese kale.

### Gene expression analysis of *FLC* homologs in kale and Chinese Kale

**Fig. 5:**
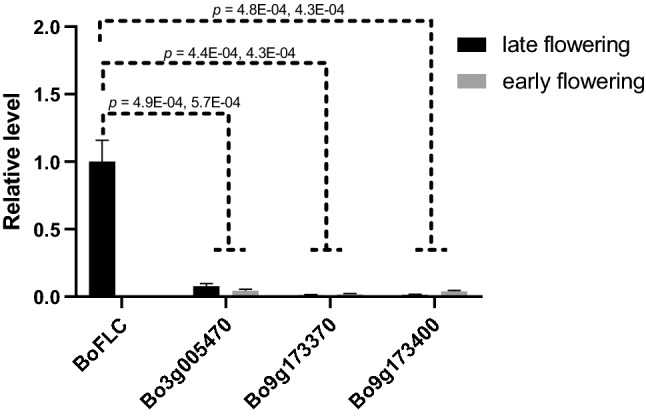
qRT-PCR of *FLC* homologs in early flowering and late flowering individuals. The statistical significances were determined using Student’s *t*-test. Error bars represent standard error

Our data indicated that although there were five *FLC* homologs in the genomes of *B. oleracea,* vernalization was determined by *BoFLC* alone. We hypothesized that the lack of functional redundancy was caused by a low gene expression of other *FLC* genes. To test this hypothesis, we analyzed the expression of three *FLC* homologs (excluding *Bo3g024250*) in individuals of F_4_ family (Fig. 5).

Interestingly, *Bo9g173370*, *Bo9g173400* and *Bo3g005470* exhibited no significant difference in expression level between early flowering individuals (without *BoFLC*) and nonflowering individuals (with intact *BoFLC*) (*t*-test; *p* = 0.64, 0.15, and 0.35). Furthermore, the expression levels of these three genes were much lower than that of *BoFLC* in late-flowering individuals. Consequently, the presence/absence of *BoFLC* determined the variation of total *FLC* expression levels, thus leading to variation on vernalization requirement. We conclude that the *FLC* homologs have no functional redundancy simply because other *FLC* genes have negligible expression.

### Retainment of absence of *BoFLC* for early bolting in Chinese kale

**Fig. 6:**
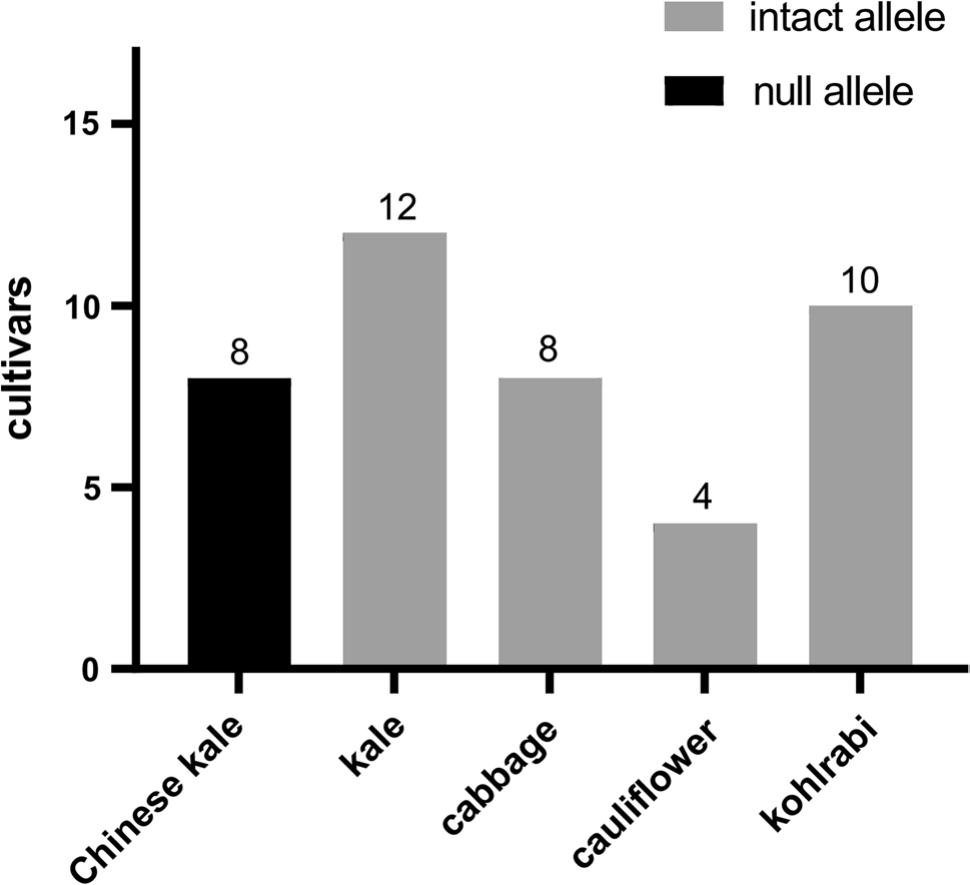
Presence/absence of *BoFLC* in different cultivars of *B. oleracea*. The y-axis represents the number of cultivars

A primer pair specific to the presence/absence polymorphism of *BoFLC* was used to genotype 42 cultivars of *B. oleracea*, including cabbage, broccoli, cauliflower, Chinese kale, kale, Brussels sprouts, and kohlrabi (supplemental dataset 2). Strikingly, all of the eight Chinese kales investigated in this study lost the *BoFLC* gene, while kale, cabbage, cauliflower, kohlrabi cultivars had the intact *BoFLC* gene (Fig. 6). This is consistent with the fact that Chinese kale is the only non-vernalization crop in *B. oleracea*. Thus, we conclude that loss of *BoFLC* occurred during breeding program with the purpose of cultivating early bolting of Chinese kale since its stem was the main harvested and consumed parts.

## Discussion

### BSA plus high-throughput sequencing facilitates cloning of genes controlling complex genetic traits

QTL mapping is commonly used to identify genetic regions controlling important traits by constructing recombinant inbred lines (RILs). RILs are usually established by several generations of inbreeding of an F_1_ population (up to F_6_ or F_7_ populations) (Li et al. 2021; Qiao et al. 2021; Xu et al. 2021). Such an inbreeding process is labor- and time-consuming. In contrast, bulked segregant analysis is a fast, economical, and efficient method for genetic analysis (Michelmore et al. 1991). High-throughput sequencing technology is now frequently used in combination with BSA to study complex genetic traits such as leaf colors and heading traits in lettuce (Su et al. 2020; Yu et al. 2020), fruit shape trait in wax gourd (Cheng et al. 2021), blossom-end rot trait in tomato (Topcu et al. 2021), grain weight in rice (Du et al. 2021), fruit spines in spinach (Liu et al. 2021). In this study, we employed BSA + RNA-seq method to identify the candidate gene *BoFLC,* which further confirmed that BSA + high-throughput sequencing could greatly facilitate the cloning of genes controlling complex genetic traits. In addition, when the candidate gene was absent in the reference genome of a certain species, syntenic region analysis of related species might contribute to the identification of the candidate gene.

### *FLC* homologs play key roles in regulating flowering time in Brassica

One previous study has identified a QTL affecting flowering time in broccoli, which was syntenic to the region of *BoFLC*. The candidate gene controlling flowering time in broccoli was predicted to be *Bol024659*, a homolog of *GRF6* in Arabidopsis (Shu et al. 2018). However, the *Bol024659* gene was located outside the 80 kb candidate region of *KVG1* in this study, therefore, this gene was excluded as the candidate gene for *KVG1*. Our data showed that the absence of *BoFLC* was responsible for non-vernalization in Chinese kale.

Previous QTL analyses of flowering time in Brassica have revealed that the *FLC* family plays a key role in regulating flowering time in *B. napus*, *B. oleracea*, and *B. rapa*. Specifically, in *B. rapa*, a naturally occurring deletion spanning exon 4 and intron 4 in *BrFLC2,* and a G/A polymorphism at the 5`splice site in intron 6 of *BrFLC1* contribute to flowering time variations (Wu et al. 2012; Yuan et al. 2009). In *B. napus*, a *Tourist*-like MITE insertion into the upstream region of the *BnFLC.A10* gene affects flowering time in rapeseed (Hou et al. 2012). In *B. oleracea*, the *BoFLC.C2* was co-localized with one QTL regulating flowering time in broccoli (Irwin et al. 2016; Okazaki et al. 2007). The above studies indicate that the polymorphisms of *FLC* homologs in Brassica affect flowering time, but those polymorphisms did not change the vernalization requirement. Our current study reveals that the absence of *BoFLC* can lead to Chinese kale flowering without vernalization. Although the *BoFLC* is the major component for controlling flowering time (vernalization) variation, the effects of other *BoFLC* homologs could not be excluded in *B. oleracea* species.

### Total expression level of *FLC* homologs controls flowering time

The role of *FLC* homologs in regulating flowering time is similar across Brassicaceae species (Leijten et al. 2018). The *B. oleracea* genome has five *FLC* homologs. The retention of *FLC* paralogs contributes to the maintenance of stoichiometric expression balance, and over-retention of the *FLC* paralogs suggests dosage-sensitivity of *FLC* family (Maere et al. 2005). If selection pressure to keep balance acts on the total gene expression level of a functionally similar gene family, the expression level of individual paralogs will fluctuate, and this effect caused by this fluctuation is compensated for by other paralogs (Birchler and Veitia 2010, 2012; Thompson et al. 2016). Total expression of all the *BnaFLC* paralogs rather than the expression of individual *BnaFLC* paralogs determine vernalization requirement in *B. napus* (Calderwood et al. 2021). In the current study, we discovered that the expression of *BoFLC* was much higher than that of its paralogs in *B. oleracea.* Thus, the *BoFLC* was decisive for the vernalization requirement. Polymorphisms were also detected in other *FLC* paralogs, and they might also affect flowering time variation but not vernalization requirement.

### Presence/absence of *BoFLC* can be used in future breeding of *B. oleracea*

*B. oleracea* includes several cultivars such as cabbage, broccoli, cauliflower, Chinese kale, kale, Brussels sprouts, and kohlrabi. The main consumed part of kohlrabi is swollen stem, and bolting stem for Chinese kale. Except for Chinese kale and kohlrabi, leaves are the main edible part of other cultivars. The loss of *BoFLC* is only detected in Chinese kale. Early flowering with no vernalization requirement is a favorable trait for Chinese kale breeding. We speculated that the 9,325-bp deletion fragment containing the *BoFLC* gene was selected for the early flowering of Chinese kale. The null allele of *BoFLC* could be introduced into other *B. oleracea* sub-species such as cabbage to shorten the reproduction time of inbred lines. If an inbred line with the null allele is crossed with an inbred line with the intact allele of *BoFLC,* its vernalization requirement can be recovered in the resulting hybrid cultivar.

## Supplementary Information

Below is the link to the electronic supplementary material.

Supplementary file1 (XLSX 17 kb)

## Declarations

### Data availability statement

The RNA-seq data supporting the results of this study are available in NCBI SRA (Sequence Read Archive, http://www.ncbi.nlm.nih.gov/sra/) repository under BioProject PRJNA743408. The gene (*BoFLC*) sequences are available in NCBI GenBank (https://www.ncbi.nlm.nih.gov/genbank/) under accession number MZ501613 in BankIt.
